# Supplementary material for: Development of an Optimized Clearing Protocol to Examine Adipocyte Subpopulations in White Adipose Tissue
Source: Methods Protoc. 2021 Jun 2;4(2):39. doi: 10.3390/mps4020039 (PMC8293430; doi:10.3390/mps4020039)
Supplement: Supplementary file 1 [file mps-04-00039-s001.zip › mps-1223619-supplementary.pdf]

SUPPLEMENTAL INFORMATION:

Development of an Optimized Clearing Protocol to Examine Adipocyte Subpopulations in White Adipose Tissue

Quyen Luong[1,2], Andreas Israel[3,4,5], Rita Sharma[1,2], Siegfried Ussar[3,4,5], Kevin Y. Lee*[1,2]

[1]Department of Biomedical Sciences, Heritage College of Osteopathic Medicine, Ohio University, Athens, OH

[2]The Diabetes Institute, Ohio University, Athens, OH

[3]German Center for Diabetes Research (DZD), Neuherberg, Germany

[4]Department of Medicine, Technical University of Munich, Munich, Germany

[5]RG Adipocytes & Metabolism, Institute for Diabetes & Obesity, Helmholtz Diabetes Center, Helmholtz Center Munich, Neuherberg, Germany

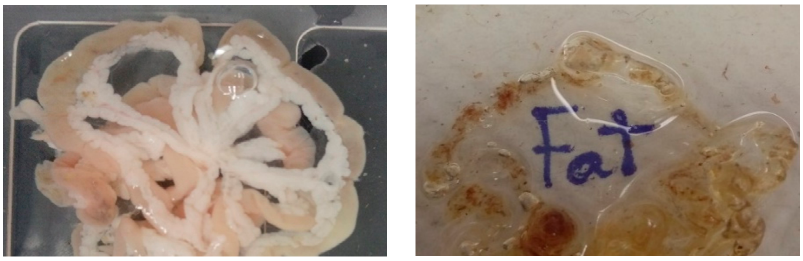

(**A**) (**B**)

Figure S1. BABB-D4 clearing of mesenteric fat. Gross histology: (**A**) pre-clearing image; (**B**) post-clearing image. A part of the intestines has been flipped to the right of the image to show the transparency of the adipose tissue.

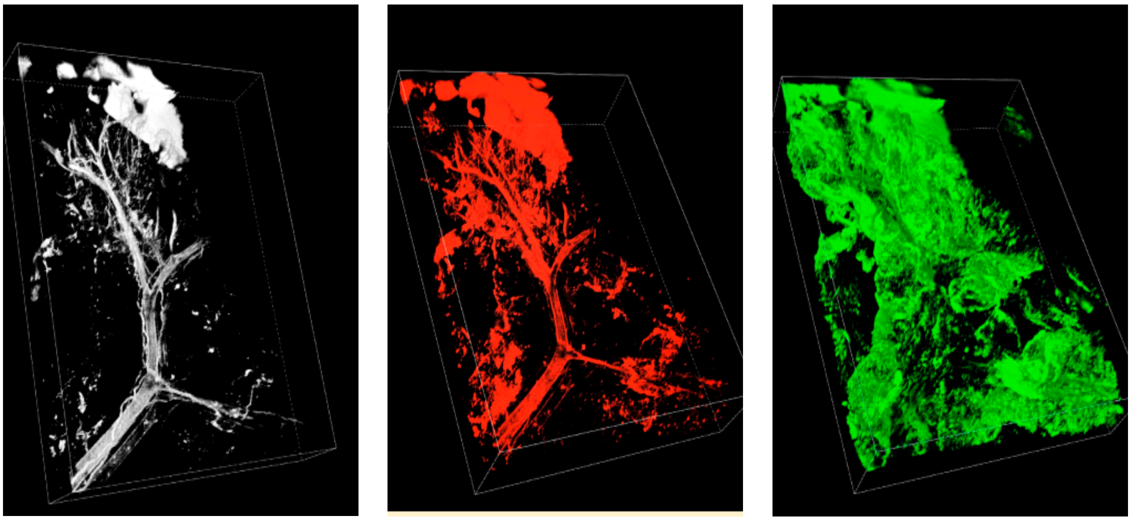

(**A**) **Sympathetic Nerves** (**B**) **Blood Vessels** (**C**) **Type-1 Adipocytes**

Figure S2. BABB-based clearing without DCM delipidation successfully labelled sympathetic nerves and blood vessels. (**A**) sympathetic nerves (anti-tyrosine kinase), (**B**) blood vessels (isolectin B4), and (**C**) Type 1 adipocytes. All pictures were taken at 100x magnification. Scale bar = 100 μm.

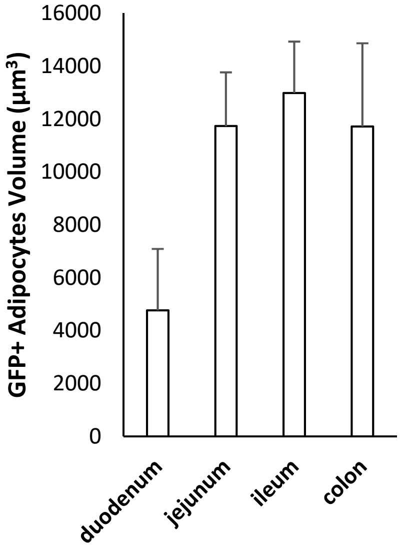

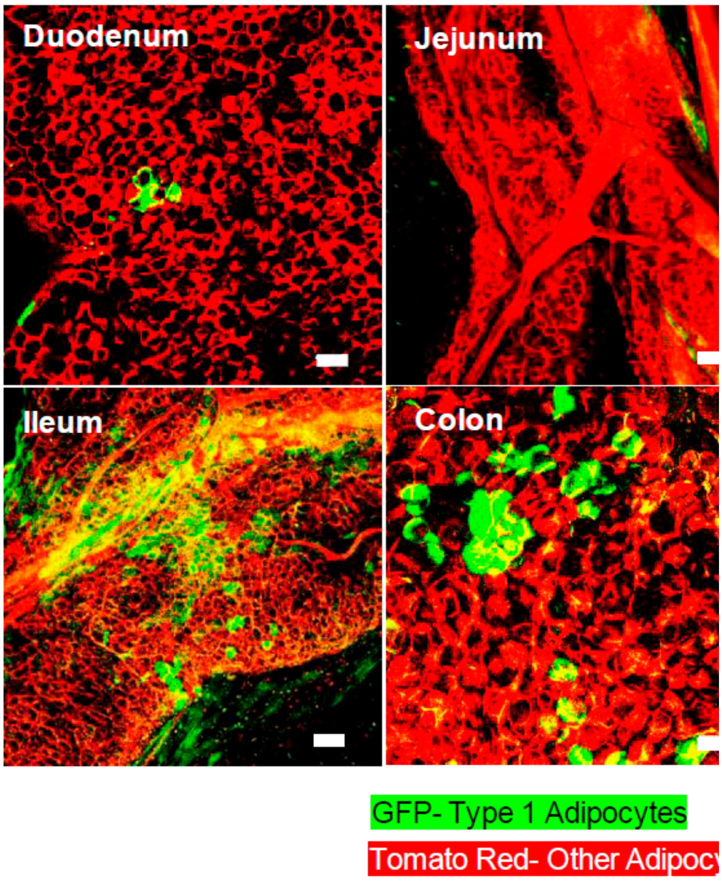

(**A**) (**B**)

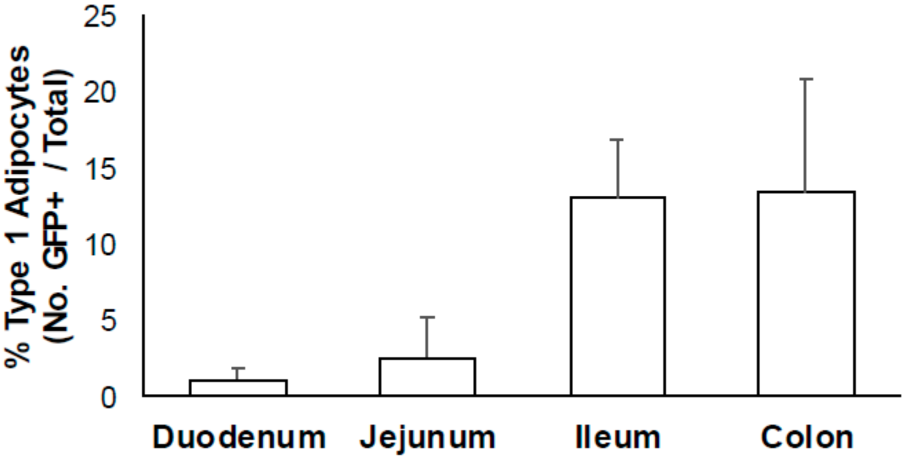

(**C**)

Figure S3. Confocal whole mount fluorescent imaging of mesenteric fat confirms findings of the tissue clearing technique as Type-1 adipocytes are associated with the ileum and colon. A) Average calculated volumes of GFP+ adipocytes in mesenteric fat associated with different intestinal regions; B) Representative confocal images of whole mount fluorescence of mesenteric fat from two 6-week-old Wt1-cre^{ERT2}/ROSA26^{mTmG} mice associated with the duodenum, jejunum, ileum, and colon; C) Quantitation of GFP+ adipocytes in each region. All pictures were taken at 100x magnification. Scale bar = 100 µm.

Kernel Density Estimation Script

The following is the Kernel density estimation script written in R Studio.

```
#Loading Perl packages
library(imager)
library(ggplot2)

#loading images
Image <-load.image('')
Control <-load.image('')

#image processing into numerical values
fat <-as.data.frame(Image)
ctrl <-as.data.frame(Control)

#because individual image is a combination of three colors (Red, Green, and Blue), blue is extracted because it does not interfere with the existing green and red fluorescence colors in the image.
cells <- fat[which(fat$cc =='3'),]
cellsctrl<-ctrl[which(ctrl$cc=='3'),]

#convert those data point back to image format using cimg
cells <- points %>% as.cimg
cellsctrl<-pointsctrl %>% as.cimg

#convert it to grayscale for easier manipulation later on
c <-grayscale(cells)
cctrl<-grayscale(cellsctrl)

#blur out the image
c <- isoblur(c,10)
cctrl<-isoblur(cctrl,2)

#convert those data point back to image format using cimg
cells <- cells %>% as.cimg
cellsctrl<-cellsctrl %>% as.cimg
```

#Mathematically speaking, local maxima and minima can be found using second order derivatives. Because an image had both x and y variables, a second order partial derivative, f''(x), such as the Hessian matrix was used. Hessian matrix is a square matrix of second-order partial derivatives function. The following equation described the Hessian matrix, where $\boldsymbol{I_{xx}}$ and $\boldsymbol{I_{yy}}$ are second partial derivatives of x and y, respectively.

$$\mathbf{det}(\boldsymbol{H}) = \boldsymbol{I_{xx}} \times \boldsymbol{I_{yy}} - \boldsymbol{I_{xy}^2}$$

```
Hdet<-with(imhessian(c),(xx*yy-xy^2))
Hdetctrl<-with(imhessian(cctrl),(xx*yy-xy^2))
plot(Hdet)

#set threshold to allow only points greater than 99.5% intensity to show up and stored as lab and
labctrl for experimental and control, respectively.
lab <- threshold(Hdet,"99.5%")%>%label
labctrl <- threshold(Hdetctrl,"99.5%")%>%label
plot(lab) #check to see if the points detected were only the GFP+ adipocytes initially present in the
image.

#convert to data frame and retrieve any data with value greater than 0 (non-background)
df<-as.data.frame(lab)%>%subset(value>0)
dfctrl<-as.data.frame(labctrl)%>%subset(value>0)
unique(df$value)
unique(dfctrl$value)

#finding center points (local maxima)
centers <-ddply(df,.(value),summarize, x=mean(x),y=mean(y))
centersctrl <-ddply(dfctrl,.(value),summarize, x=mean(x),y=mean(y))

#plot center points with original blob plots
plot(c)
with(centers,points(x,y,col="red"))
plot(cctrl)
with(centersctrl,points(x,y,col="red"))

#pixels for those center points for KDE analysis were retrieved; retrieve columns 2 and 3 from the
previous data
xypixels <- centers[,c(2,3)] #GFP+ adipocytes collected
xyctrl<-centersctrl[,c(2,3)] #control points

#Statistical test
library(ks)

#GFP+ cells vs all control cells and rounding to three digits of significant digits
w<-signif(kde.test(x1=xypixels,x2=xyctrl)$pvalue,digits=3)

#plotting images
m = ggplot(xypixels, aes(x=x,y=y)) +
  stat_density2d(aes(alpha=..level..,fill=..level..), size=2,bins=20, geom="polygon")+
scale_fill_gradient(low = "yellow", high = "red") +
  scale_alpha(range = c(0.01, 0.8), guide = FALSE)+
```

```
    geom_density2d(colour="blue", bins=10)+
    geom_point(data=xypixels)+
    guides(alpha=FALSE) + xlim(0, 1900) + ylim(0, 1400)+
    ggtitle("Mesenteric fat")+
    annotate("text",x=1500,y=200, label='p-value=')+
    annotate("text",x=1500,y=100,label=w)
plot(m)
```
